# COVID-19 Pandemic on Fire: Evolved Propensities for Nocturnal Activities as a Liability Against Epidemiological Control

**DOI:** 10.3389/fpsyg.2021.646711

**Published:** 2021-03-22

**Authors:** Marco Antonio Correa Varella, Severi Luoto, Rafael Bento da Silva Soares, Jaroslava Varella Valentova

**Affiliations:** ^1^Department of Experimental Psychology, Institute of Psychology, University of São Paulo, São Paulo, Brazil; ^2^English, Drama and Writing Studies, University of Auckland, Auckland, New Zealand; ^3^School of Psychology, University of Auckland, Auckland, New Zealand; ^4^Center for Science Communication and Education Studies, Department of Genetics, Evolution, Microbiology, and Immunology, Institute of Biology, University of Campinas, Campinas, Brazil

**Keywords:** pandemic (COVID-19), health measures, non-compliance behavior, chronotype (morningness–eveningness), evolutionary mismatch hypothesis, evolutionary psychology, life history theory, non-adherence

## Abstract

Humans have been using fire for hundreds of millennia, creating an ancestral expansion toward the nocturnal niche. The new adaptive challenges faced at night were recurrent enough to amplify existing psychological variation in our species. Night-time is dangerous and mysterious, so it selects for individuals with higher tendencies for paranoia, risk-taking, and sociability (because of security in numbers). During night-time, individuals are generally tired and show decreased self-control and increased impulsive behaviors. The lower visibility during night-time favors the partial concealment of identity and opens more opportunities for disinhibition of self-interested behaviors. Indeed, individuals with an evening-oriented chronotype are more paranoid, risk-taking, extraverted, impulsive, promiscuous, and have higher antisocial personality traits. However, under some circumstances, such as respiratory pandemics, the psychobehavioral traits favored by the nocturnal niche might be counter-productive, increasing contagion rates of a disease that can evade the behavioral immune system because its disease cues are often nonexistent or mild. The *eveningness epidemiological liability hypothesis* presented here suggests that during the COVID-19 pandemic, the evening-oriented psychobehavioral profile can have collectively harmful consequences: there is a clash of core tendencies between the nocturnal chronotype and the recent viral transmission-mitigating safety guidelines and rules. The pandemic safety protocols disrupt much normal social activity, particularly at night when making new social contacts is desired. The SARS-CoV-2 virus is contagious even in presymptomatic and asymptomatic individuals, which enables it to mostly evade our evolved contagious disease avoidance mechanisms. A growing body of research has indirectly shown that individual traits interfering with social distancing and anti-contagion measures are related to those of the nocturnal chronotype. Indeed, some of the social contexts that have been identified as superspreading events occur at night, such as in restaurants, bars, and nightclubs. Furthermore, nocturnal environmental conditions favor the survival of the SARS-CoV-2 virus much longer than daytime conditions. We compare the eveningness epidemiological liability hypothesis with other factors related to non-compliance with pandemic safety protocols, namely sex, age, and life history. Although there is not yet a direct link between the nocturnal chronotype and non-compliance with pandemic safety protocols, security measures and future empirical research should take this crucial evolutionary mismatch and adaptive metaproblem into account, and focus on how to avoid nocturnal individuals becoming superspreaders, offering secure alternatives for nocturnal social activities.

## Introduction

Everything in pandemics is stamp collection except in the light of evolution. Evolutionary approaches to human behavior have the potential of uncovering hidden patterns and deep roots of many seemingly disparate findings (DeBruine, 2009; Gentle and Goetz, 2010; Stephen et al., 2014; Buss, 2020). And the context of pandemics, particularly the current COVID-19 context, is no different (Arnot et al., 2020; Dezecache et al., 2020; Seitz et al., 2020; Troisi, 2020; Luoto and Varella, 2021). Besides the evolved tendencies to respond to cues of diseases avoiding contagion (i.e., behavioral immune system; Shook et al., 2020; Stevenson et al., 2021), and to deal with pathogens (i.e., immune system; Krams et al., 2020), there are many other evolutionary factors that play a role in a pandemic situation (Arnot et al., 2020; Seitz et al., 2020). For instance, the evolved sex differences in psychological tendencies might help to explain why women are more careful and comply with safety measures more than men, and importantly why female leaders are more successful in minimizing deaths in a pandemic context than male leaders (Luoto and Varella, 2021). Further insight can be acquired via the evolutionary mismatch hypothesis (Lloyd et al., 2011; Li et al., 2018), in which evolved tendencies in response to specific ancestral contexts become a burden in current environments. Besides explaining the contemporary proliferation of mental health problems (Rantala et al., 2018, 2019, 2021), the evolutionary mismatch framework (Li et al., 2018) can also offer a heuristic and promising perspective into some maladaptive behaviors in the COVID-19 pandemics (Seitz et al., 2020).

This hypothesis and theory article aims to explore one instance of how evolutionary mismatch could help to explain disparate findings related to the current COVID-19 pandemic by uncovering a crucial factor possibly affecting the spread of the virus: the nocturnal psychobehavioral core of non-compliance to safety measures. In doing so, we suggest to take into account nocturnal risk-taking individuals in public health communications to improve compliance with virus-mitigating measures in order to possibly decrease the rate of contagion and superspreading events. However, application of behavioral science as public policy always needs to be made with extreme care and caution (IJzerman et al., 2020). We hope to both promote new discoveries and improve public policies, but also inspire scientists and policy-makers to use the advantages of an evolutionary perspective to help attenuate the consequences of the COVID-19 pandemic.

The main new hypothesis put forward here is *the eveningness epidemiological liability hypothesis*, which posits that evolved propensities for nocturnal activities, though adaptive in their own evolved context, have currently become a dangerous liability against epidemiological control. Although there were frequent and recurrent disease outbreaks in *Homo sapiens’* ancestral environment(s), the current SARS-CoV-2 virus seems to evade the evolved contagious disease avoidance tendencies because it makes individuals contagious before being symptomatic (e.g., Tindale et al., 2020) and it results in very mild or no symptoms in most individuals (Grant et al., 2020; Huff and Singh, 2020). This mismatch could be somewhat mitigated in Asian people, as it has been hypothesized that high levels of visceral adiposity in Southeast Asians might be an evolutionary response against previous encounters with coronaviruses in this region, but that Western populations lack this evolutionary adaptation against coronaviruses and could therefore be biologically more vulnerable to SARS-CoV-2 (Krams et al., 2020, 2021). Furthermore, there were probably few socially recommended restrictive virus-mitigating protocols and pro-health measures ancestrally, at least to the same extent that is now possible with the global proliferation of scientific information used to mitigate the COVID-19 pandemic.

### The Relevance of Chronotypes to the COVID-19 Pandemic

Despite these evolutionarily novel societal protocols, evening-oriented individuals and others with a similar risk-taking psychobehavioral profile (e.g., Corpuz et al., 2020; Luoto and Varella, 2021) still tend to behave according to their evolved predispositions, creating an evolutionary mismatch (cf. Li et al., 2018; Ackerman et al., 2020; Dezecache et al., 2020; Seitz et al., 2020). Nocturnal individuals (i.e., those with a chronotype higher on eveningness, night-owls; Byrnes et al., 1999) tend to be more risk-taking, extraverted, impulsive, promiscuous, and have more pronounced Dark Triad personality profiles (i.e., psychopathy, Machiavellianism, narcissism) (cf. Digdon and Howell, 2008; Russo et al., 2012; Jonason et al., 2013; Fabbian et al., 2016; Sheaves et al., 2016; Díaz-Morales et al., 2019). Although evening-oriented individuals are also more intelligent (e.g., Randler, 2017), more capable of perceiving and understanding emotion (Stolarski and Jankowski, 2015), open to experience (Randler et al., 2017), and visually creative (Giampietro and Cavallera, 2007) than morning-oriented individuals, a great portion of their profile aligns with the profile(s) so far found in individuals known to be likely to break COVID-19 pro-health rules. There is still a lack of direct and systematic evidence linking chronotype and compliance with pandemic safety measures, and the high similarity of both profiles deserves more attention from behavioral scientists and psychologists. We hope that by proposing our hypothesis, we inspire new research and stimulate the design of a more effective and persuasive communication of anti-contagion public policies.

The crucial task of reducing contagion and viral transmission is as much a problem of psychology and behavioral science as it is of epidemiology and virology (Kantor, 2020; Smith and Gibson, 2020; Van Bavel et al., 2020). Psychological factors play an essential role for the success of risk communication, endorsement of vaccines and antiviral therapies, compliance with hygiene practices and social distancing (Taylor, 2019). Many fields of psychology and social sciences have been mobilized to help create and promote health safety measures in order to slow the contagion rate of SARS-CoV-2 (Smith and Gibson, 2020; Van Bavel et al., 2020). About a third of individuals are non-adherent to preventive measures (e.g., Nzaji et al., 2020). Because there are some individuals and contexts that are more likely to generate superspreading events, and the available evidence shows that a ∼10% minority of superspreaders account for a large majority of the infections (Chang et al., 2020), even a small proportion of non-compliers can be very influential in worsening the transmission rate and throwing more gasoline into the pandemic fire (Wong and Collins, 2020).

First, we review the evolution of the use of fire that promoted the expansion of the nocturnal niche in humans (see section “The use of fire expanded the night-time activities”). The antiquity and recurrent fire control together with the difference between non-human ape and human sleep patterns indicates that over hundreds of millennia, humans have become gradually more nocturnal. Then, in order to refine the evolutionary consequences of that nocturnal expansion, we identify the key adaptive challenges that ancestral humans recurrently faced during night-time (i.e., its inherent imminent danger, peak tiredness, and easy concealment of identity) (see section “Night-time adaptive challenges”). Each of these challenges may have selected for an increased psychological tendency that together compose a part of the eveningness chronotype (i.e., the night-owl profile). Sexual selection and fast life history strategy probably play an important role in composing the eveningness chronotype (see section “Sexual selection for eveningness and fast life history strategies”).

Further, we dissect the layers of related evolutionary mismatches and conflicting adaptive problems and show how there is a clash of core tendencies between the evening-oriented profile and the COVID-19 health safety protocols (see section “Evolutionary mismatches during the COVID-19 pandemic”). Then, we show that the available evidence on the individual-level predictors of non-compliance with the pandemic safety measures is very similar to that of the eveningness chronotype. We also show that some of the social contexts that have been identified as superspreading events occur during the night. Moreover, the new coronavirus is able to survive much longer in aerosols at night (see section “Eveningness epidemiological liability hypothesis”).

We discuss the implications of the eveningness epidemiological liability hypothesis for explaining non-compliance with the pandemic safety measures via the ability of this multilevel evolutionary approach to organize disparate findings and open new lines of inquiry. Sexual selection and life history theory are the broader evolutionary dynamics within which we contextualize this model. We close with tentative suggestions for public health campaigns to target evening-oriented individuals, and for public authorities and private initiatives to promote, after careful consideration, epidemiologically secure alternatives for at least a part of their nocturnal social activities.

## The Use of Fire Expanded the Night-Time Activities

Vervet monkeys show less frequent predator-related behaviors (e.g., alarm calls) in recently burned habitats, suggesting that predators are less common in these areas, which in turn might explain survival advantages for human ancestors to initially pay attention to and follow the natural fires for defense purposes (Herzog et al., 2020). Humans have such an old and diversified contact with fire that they are considered the pyrophilic primate (Parker et al., 2016). The proposition that early hominins in Africa sporadically and opportunistically used fire around 1.6 mya (Roebroeks and Villa, 2011; Gowlett, 2016) matches the consequent onset of digestive adaptations for cooked food, e.g., gracile jaw and teeth (Wrangham, 2017), and the evidence for increased out-of-Africa migration toward colder locations (Gowlett, 2006; Brown et al., 2009). After the initial opportunistic phase, in which ancestral hominins were adapting to progressively dry, hence fire-prone environments (Parker et al., 2016), came a transitory phase that led to the habitual use of fire (Gowlett, 2016). Habitual use of fire dates to around 400–300 kya in Neanderthals and somewhat later in modern humans (Roebroeks and Villa, 2011).

Fire brought many benefits to our ancestors, such as heat and light, protection against predators and other nocturnal threats, sterilization, preservation, flavorization, easily digestible cooked food, and even aid in the manufacturing of tools (Wrangham, 2009; Dunbar and Gowlett, 2014). These advantages of fire control set the stage for socioecological (Dunbar and Gowlett, 2014), cognitive (Twomey, 2013), and cultural (Wiessner, 2014; Mithen, 2019) evolutionary change.

Among many other changes, the control of fire represents an important evolutionary factor toward increasing human nocturnal behaviors. Occasional nocturnal behavior is found in other apes such as in chimpanzees (Merker et al., 2015; Tagg et al., 2018) and in orangutans (Samson et al., 2014). Still, evidence indicates that modern humans did not merely retain the basic ape-like occasional nocturnal behaviors, but significantly expanded their nocturnal lifestyle. Modern humans in fact exhibit a derived sleep pattern that is different from the other apes. Human sleep is shorter, deeper, and exhibits more events of REM sleep which might have better maintained high levels of cognitive performance and freed nocturnal time for awake activities (Samson and Nunn, 2015; Nunn et al., 2016). Indeed, in humans a considerable proportion of the population presents a stable and moderately heritable predisposition for night-time activity (e.g., Vink et al., 2001; Hur, 2007).

The use and control of fire in humans is ancient and recurrent (Roebroeks and Villa, 2011; Gowlett, 2016) enough to have acted as a strong selective pressure (Twomey, 2013; Dunbar and Gowlett, 2014; Wrangham, 2017). Aligned with the difference between ape and human sleep patterns (Samson and Nunn, 2015; Tagg et al., 2018), fire use provides compelling evidence indicating that humans indeed have expanded their nocturnal niche over hundreds of millennia (cf. Piffer, 2010; Putilov, 2014). Together with fire, many other selective pressures, such as increased predation risk in terrestrial environments, threats from intergroup conflict, and benefits arising from increased social interaction may have converged to expand evening-orientation in humans (Ponzi et al., 2015b; Samson and Nunn, 2015). Increased night-time activity may have amplified existing psychological variation in our species in response to key night-time adaptive challenges (cf. Marvel-Coen et al., 2018).

## Night-Time Adaptive Challenges

The expansion into the nocturnal niche enabled by the use of fire posed new specific and recurrent adaptive challenges to ancient hominins, related to its inherent dangerousness, peak tiredness, and easy concealment of identity. Independently or in combination, these key features were probably relevant selective pressures that the ancestral populations faced in order to survive and benefit from the nocturnal niche. Particularly, the existing specific psychological variation that might have been adaptively reoriented and reorganized to deal with the key night-time challenges would have been effectively related to the correspondent variation in human chronotype.

*Chronotype* reflects individual underlying circadian rhythm (Jones et al., 2019). It is a psychophysiological concept that captures the natural individual variation in circadian well-functioning/preferences between ‘morning types’ all the way to ‘evening types’ on a continuous spectrum of sleep timing (Byrnes et al., 1999; Vink et al., 2001; Hur, 2007; Jones et al., 2019). There are morning lark individuals (i.e., with a chronotype higher on morningness, early to go to bed and early to wake up), intermediate individuals, and night-owl individuals (i.e., with a chronotype higher on eveningness, late to bed, late to wake up) (Byrnes et al., 1999; Vink et al., 2001; Hur, 2007; Jones et al., 2019).

### Dangerousness

Night-time is universally dangerous and mysterious, because the diminished light makes it hard to foresee danger; what is more, there are typically fewer people around to alert and protect those who stay up late, thus increasing vulnerability. Darkness increases uncertainty, tension, and anxiety, which facilitates the acoustic startle reflex (Grillon et al., 1997), and leads to realistic/imagined fears of the dark (i.e., Nyctophobia) (Levos and Zacchilli, 2015), so much so that staring at a stationary point of light in the dark activates motion perception, generating an autokinetic illusion (i.e., the illusory movement of the light; Riedel et al., 2005), which is typical of agents.

There is an evolved and strategic shift in thresholds for signal detection when we feel in danger. Natural selection tends to select the less costly error particularly when the cost of not seeing a real threat (i.e., false negative) is greater than seeing an illusion of threat (i.e., false positive) (Haselton and Buss, 2000; Haselton and Nettle, 2006). Indeed, once in fear, the individual needs less evidence to trigger the threat reaction; thus, a higher rate of false alarms will be perceived, protecting against the cost of not perceiving a real threat (Tooby and Cosmides, 2008).

Because of the extended nocturnal niche, we might have developed a powerful and paranoid imagination, an evolved tendency to overestimate threats, purposes, and agency, particularly those stemming from enemies (Varella, 2018). In fact, Sheaves et al. (2016) showed that the eveningness chronotype is directly related to psychiatric symptoms of paranoia, hallucinations, anxiety, and depression. Large-sample genome-wide association studies have found shared underlying genetic pathways between eveningness chronotype and schizophrenia (Jones et al., 2016; Lane et al., 2016), and that being a night person is causally associated with worse mental health (i.e., less subjective well-being and more major depression) (Jones et al., 2019). Paranoid ideation and schizotypy predict conspiracy beliefs independent of sex (Darwin et al., 2011). Paranoia and belief in conspiracy are indeed positively correlated (Imhoff and Lamberty, 2018). Importantly, the anxiety and uncertainty experienced during epidemics and pandemics also tend to boost conspiracy thinking (Smallman, 2015, 2018; Taylor, 2019; Desta and Mulugeta, 2020).

At night, chances are higher to suffer from predator attacks (e.g., lions) in small-scale societies (Packer et al., 2011), or personal contact crimes in industrial societies (Averdijk and Bernasco, 2015). A similar level of dangerousness, if not worse, was possibly recurrent at night during ancestral times, given that the level of mortality in intergroup conflicts was substantial (Bowles, 2009), and ancestral hominids were preyed on by several large diurnal and nocturnal felines (Lee-Thorp et al., 2000; Treves and Palmqvist, 2007). This adaptive challenge could have selected for evening-oriented individuals to be more risk-taking and sensation-seeking, for them to be willing to face the elevated nocturnal risks. By facing the nocturnal risks, they could have served as guards for their significant others asleep (i.e., kin selection; Gardner et al., 2011), or they could have displayed to potential mates their survival qualities despite the handicap of vulnerability in the darkness (i.e., costly signaling in sexual selection; Grafen, 1990; Zahavi and Zahavi, 1999). Indeed, empirical studies indicate that chronotype variation leads to sleep asynchrony which function as protective night-time vigilance by having evening-oriented individuals as sentinels (Samson et al., 2017), and the sexual selection have also influenced the eveningness chronotype (Piffer, 2010; Randler et al., 2012b; Putilov, 2014; Ponzi et al., 2015a, b). Moreover, for protective reasons, the *herd principle of ‘security in numbers,’* which is for instance used as rows of nearby sleepers to insure night-time safety by Aboriginal settlements (Musharbash, 2013), as well as the equivalent *many eyes hypothesis* that is a predator-avoidance strategy which facilitates mixed-species bird flocking (Krams et al., 2020), could have been recurrently used in ancestral times, so much so that it would have selected for evening-oriented individuals to be gregarious, prioritizing social agglomerations. Human social conversational sounds could have indeed frightened ancestral predators, since large carnivores avoid human voices (Suraci et al., 2019). The nocturnal agglomeration and profusion of vocal sounds would have complemented the predator deterrent effect of fire (Wrangham, 2009; Dunbar and Gowlett, 2014).

Indeed, independent of sex and personality, individuals scoring higher on eveningness chronotype report higher risk-taking (Ponzi et al., 2014), sensation-seeking, and impulsivity (Russo et al., 2012; Antúnez et al., 2014). Men are, on average, more prone to risk-taking than women (Byrnes et al., 1999; Archer, 2019), and men aged 40 years and below are more nocturnal than women (Piffer, 2010; Fischer et al., 2017). Evening-oriented individuals also tend to be extroverted and have high openness to experience (Lipnevich et al., 2017), thus exhibiting the sociability to guarantee the ‘security in numbers’ through human vocal sounds around the fireplace, as currently done at the firelight talk among the Bushmen (Wiessner, 2014).

### Peak Tiredness

During night-time, after the working hours, individuals are at their peak tiredness. This disrupts self-regulation and predisposes to impulsive behaviors, succumbing to immediate pleasures, such as cravings for food (Sevincer et al., 2016), alcohol and club/party drug use (Millar et al., 2019), and sexual intercourse (Refinetti, 2005; Jocz et al., 2018). Indeed, evening-oriented individuals have lower self-control (Digdon and Howell, 2008), which mediates their higher present orientation and instant gratification (Milfont and Schwarzenthal, 2014), are more impulsive (Cross, 2010), are more hedonistically present-oriented (Nowack and Van Der Meer, 2013; Stolarski et al., 2013; Borisenkov et al., 2019), use more alcohol and club/party drugs (Millar et al., 2019), and tend to smoke more (Randler, 2008; Wittmann et al., 2010; Patterson et al., 2016) than other chronotypes. Males exhibit lower self-regulatory capacities (Tetering et al., 2020), consume more alcohol (Salvatore et al., 2017), and are more nocturnal than females (Piffer, 2010; Fischer et al., 2017). However, women prefer to have sex later in the day than men (Jocz et al., 2018).

### Identity Concealment

The lower visibility at night compromises individual recognition, thus favoring the concealment of identity which disinhibits behaviors previously repressed due to social desirability (Zhong et al., 2010; Hirsh et al., 2011). This sense of privacy lowers the risk for social reputational damage because it enables individuals to better control of external social interferences and manage the flow of information they emit (Klopfer and Rubenstein, 1977). This adaptive challenge, among others, could have selected for evening-oriented individuals to be more willing to engage in activities that go against social desirability, such as promiscuity, aggression, rule-breaking behavior, self-interested remorseless, and interpersonal manipulation. In effect, evening-oriented individuals are more prone to casual sex, cross-culturally (Cross, 2010; Piffer, 2010; Ponzi et al., 2015b; Matchock, 2018; Díaz-Morales et al., 2019), have a faster life history strategy (Ponzi et al., 2015b; Marvel-Coen et al., 2018), have first intercourse at an earlier age (Kasaeian et al., 2019), have higher tendencies toward aggression (Schlarb et al., 2014), exhibit higher rule-breaking behavior (Merikanto et al., 2017), and have higher Dark Triad personality traits, that is, Machiavellianism (e.g., exploitative behaviors), narcissism (e.g., self-orientation), and subclinical psychopathy (e.g., lack of empathy and remorse) (Jonason et al., 2013). Men, the more nocturnal sex (Piffer, 2010; Fischer et al., 2017), also exhibit higher propensity to casual sex (Schmitt, 2005; Hughes et al., 2020), higher aggression (Archer, 2004, 2019; Cross, 2010), and higher Dark Triad personality traits (Furnham et al., 2013).

Biological sex (male/female) is relevant in this analytical context: because males are more nocturnal than females, any other sex-differentiated trait favoring males would also appear in evening-oriented individuals. This does not happen with extraversion, which is related to eveningness (Lipnevich et al., 2017), and is higher in females (Schmitt et al., 2008). Moreover, evidence indicates that the nocturnal profile is not simply derived from or due to sex *per se*. In some cases, the relationship between a given chronotype and another trait is independent of sex (e.g., Ponzi et al., 2014), and of sex and age (e.g., Milfont and Schwarzenthal, 2014). In other instances, chronotype is the factor that slightly mediates the effect of sex on a given trait, not the other way around (Rahafar et al., 2017; Gowen et al., 2019). The majority of studies either control for sex (e.g., Merikanto et al., 2017), or conduct analyses separately for each sex. Hence, all this indicates that beyond sex, chronotype *per se* is an important factor for tracking intrasexual variation in psychobehavioral predispositions.

In summary, chronotype is indeed related to solving most of these nocturnal adaptive challenges. These psychological solutions and responses to the expansion of the nocturnal niche (i.e., paranoia, risk-taking, sensation-seeking and impulsivity, extraversion, lower self-control, promiscuity, aggression, rule-breaking, and dark personality traits) correspond to the evolved nocturnal profile (cf. Fabbian et al., 2016), a constellation of propensities tuned to nocturnal activities which have been adaptively helping humans to cope and benefit from the night-time for millennia. As noted earlier, not all facets of the nocturnal profile are socially undesirable: when compared with morning-oriented individuals, evening-oriented individuals exhibit higher general and emotional intelligence (e.g., Stolarski and Jankowski, 2015; Randler, 2017), are more open to experience (Randler et al., 2017), and visually creative (Giampietro and Cavallera, 2007). The stronger nocturnal sexual selection might also explain why they have high expression of traits such as intelligence, openness, and creativity which are desired in mate choice (Stone et al., 2012).

## Sexual Selection for Eveningness and Fast Life History Strategies

As applied in psychological research (Del Giudice et al., 2016; Nettle and Frankenhuis, 2020), life history theory offers a meta-theory (Hertler et al., 2018) and a mid-level established framework to interpret mainly childhood experiences, trait covariation, and individual differences in allocation of evolutionarily relevant resources (Del Giudice et al., 2016; Krams et al., 2019; Nettle and Frankenhuis, 2020). Organisms have limited amounts of time, energy, and resources at their disposal, that need to be allocated among competing demands. This leads to well-documented tradeoffs between different life history domains, most notably investing in survival (i.e., growth, self-maintenance, and immunity) or reproduction (i.e., short-term mating and competition vs. long-term mating and parenting) (Krams et al., 2019; Luoto et al., 2019; Laskowski et al., 2020; Lauringson et al., 2020; Rubika et al., 2020).

In combination with viability selection pressures (i.e., survival), part of the evidence compiled above also supports the influence of sexual selection on the evening-oriented chronotype (cf. Piffer, 2010; Randler et al., 2012b; Putilov, 2014; Ponzi et al., 2015a, b). More generally, the findings presented reflect a well-established ‘fast’ or ‘slow’ pattern of individual-level life history variation (Del Giudice et al., 2016; Del Giudice, 2020; Nettle and Frankenhuis, 2020), namely, a tradeoff between investing in immediate rewards and short-term mating vs. in parenting, longevity and health (Sherman et al., 2013; Ponzi et al., 2015b; Marvel-Coen et al., 2018). This evolved nocturnal profile contains elements considered to represent adaptive implementations of a fast life-history strategy (Ponzi et al., 2015b; Marvel-Coen et al., 2018), which prioritizes risky sexual behaviors and short-term rewards over pandemic-mitigating measures and health behaviors (Sherman et al., 2013; Luoto et al., 2019; Arnot et al., 2020; Corpuz et al., 2020). Men with higher symptoms of paranoid ideation tend to express faster life history traits, including a tendency toward casual sex (Kahl et al., 2020). Women find male risk takers over risk avoiders more attractive for short-term mating, and the opposite for long-term relationships (Sylwester and Pawłowski, 2011). Individuals high on sensation seeking have more casual sex partners (Penke and Asendorpf, 2008; Luoto et al., 2019). Extroverted men obtain a higher mating success (Randler et al., 2012b). Individuals with higher aggression tendencies have more casual sex partners (Cross, 2010). Individuals with higher Dark Triad personality traits have more casual sex partners, higher mating effort, and lower parenting effort (Valentova et al., 2020).

Besides the connection between each aspect of the nocturnal profile with mating success, promiscuity, and fast life history, these traits are also directly related to the evening-orientation, as we have established in the previous section (Ponzi et al., 2015b; Marvel-Coen et al., 2018). Evening-oriented individuals cross-culturally exhibit higher tendencies for short-term mating and, indeed, have more casual sex (Cross, 2010; Piffer, 2010; Gunawardane et al., 2011; Ponzi et al., 2015b; Matchock, 2018; Díaz-Morales et al., 2019). Evening-type men are more flirtatious in the later part of the day (Gunawardane et al., 2011), have a higher propensity to stay out late (Randler et al., 2012b), and have higher intrasexual competition tendencies (Ponzi et al., 2015a). Evening-orientation is related to higher testosterone in men (Randler et al., 2012a), and testosterone also coordinates life history strategies across species (Luoto et al., 2019). Hence, the evening-oriented chronotype has been considered to be implementing a fast life history strategy (Ponzi et al., 2015b; Marvel-Coen et al., 2018).

All this nocturnal mating action likely results from creation of an evening lek for displaying various behaviors advertising desirable qualities through courtship (Piffer, 2010; Putilov, 2014). The primary mating ground of most modern humans (besides online dating) seems to be dancing bars and nightclubs, where people flirt and dance using their bodies to negotiate space (Nofre et al., 2017), and display sexually desirable qualities to find sexual partners (e.g., Röder et al., 2016). Correspondingly, there is a 50% increase of coupled individuals leaving the nightclub as compared to those entering it (Mannion et al., 2009). There is a distinction in mate search locations between the short-term and long-term niches, and the short-term niche includes many night-time activities, such as bars, nightclubs, parties, dance clubs, weddings, concerts, and fraternity parties (Jonason et al., 2015). The use of short-term mating locations is positively related with short-term mating orientation. And there is no sex difference in the conceptualization of which locations constitute appropriate short-term or long-term mating niches (Jonason et al., 2015).

Indeed, as a result of this temporal mating niche differentiation, individuals with a similar chronotype tend to mate assortatively (Randler and Kretz, 2011). This homogamy could be the evolutionary process promoting trait covariation (cf. Štěrbová and Valentova, 2012; Varella et al., 2012; Štěrbová et al., 2017; Conroy-Beam et al., 2019; Nishi et al., 2020) between the eveningness chronotype and the constellation of nocturnal psychobehavioral activities. For instance, behavioral genetic evidence has shown that 80% of the positive relationship between eveningness chronotype and externalizing behaviors (i.e., aggression and rule-breaking behavior) is accounted for by shared genetic influences between both tendencies, i.e., chronotype and antisocial tendencies (Barclay et al., 2011). The promiscuous short-term orientation in addition to assortative mating stressed here might better explain the high mating success of eveningness-oriented individuals than the degree of mate choosiness (cf. Staller and Randler, 2021).

The increased nocturnal sexual activity, including short-term mating-orientation and intrasexual competition, strengthened the pressure of sexual selection acting during nocturnal social contexts (cf. Piffer, 2010; Putilov, 2014). These factors converge and relate to the evidence that human sleep patterns present two hallmarks of a sexually selected trait. First, men exhibit shorter sleep and a more evening-oriented chronotype (Piffer, 2010; Randler, 2016; Fischer et al., 2017). Second, the ontogenetic phase immediately after puberty which is most typical of mating effort (i.e., late adolescence and young adulthood) is when individuals are most evening-oriented, and the sex difference fades during women’s menopause (Piffer, 2010; Randler et al., 2012b; Fischer et al., 2017).

## Evolutionary Mismatches During the COVID-19 Pandemic

A careful analysis of the ancestral versus modern, the recurrent versus rare, and convergent versus conflicting facets of selection pressures offers a complex and detailed picture of the relationship between eveningness chronotype and non-compliance with virus-mitigating public health protocols. Evolutionary mismatches have a variety of configurations, causes, and outcomes (Li et al., 2018; Rantala et al., 2019, 2021). They can be caused by a natural or human-made change in the environment that disrupts the fit between evolved adaptation and its respective ancestral recurrent selective regime (Li et al., 2018). The environmental change can be forced or hijacked, impacting an adaptation’s specific input, its existence, intensity, or by replacement of the original input by a similar one (Li et al., 2018). The consequences of mismatches can be undesirable or desirable for the individual, and they can have decreasing or increasing effects on fitness (Li et al., 2018). Furthermore, because we are dealing with two different domains (namely, chronotype, and disease avoidance) there is the possibility of convergent or conflicting selection pressures between the domains. Conflicting adaptive problems constitute an *adaptive metaproblem* (Al-Shawaf, 2016; Rantala et al., 2019). In contrast, the alignment of adaptive problems can be said to constitute an *adaptive problem-merging*. The incongruence and/or congruence between different adaptive problems can be ancestral or new, recurrent or rare. Thus, although they are independent processes, evolutionary mismatches and interaction between different adaptive problems relate to each other.

### Mismatch #1: Absence of Disease Cues Enabling the New Coronavirus to Evade Our Evolved Pathogen Avoidance Defenses

Contagious disease outbreaks and epidemics were a recurrent aspect of human ancestral environments (Fumagalli et al., 2011; Deschamps et al., 2016), so much so that beyond our evolved immune system, a growing literature has emphasized the existence and special features of the behavioral immune system (BIS) as a constellation of proactive and reactive tendencies promoting contagion avoidance (Ackerman et al., 2018, 2020; Troisi, 2020). Direct and indirect cues of diseases are relevant inputs to trigger the touch avoidance and distancing reactions of the BIS (Miller and Maner, 2012; Ackerman et al., 2018). Other highly social species also have evolved contagion avoidance reaction toward conspecifics with contagious diseases (e.g., Loehle, 1995; Behringer et al., 2006). Hence, it would be expected that humans activated similar pathogen avoidance mechanisms during the current pandemic times (cf. Troisi, 2020; Stevenson et al., 2021). Many individuals are indeed triggered by the current epidemiological threat and are socially distancing to avoid contagion (Makhanova and Shepherd, 2020), particularly women (e.g., Galasso et al., 2020), who are, on average, more disgust-sensitive and anxious about contagion (Druschel and Sherman, 1999; Fleischman, 2014; Luoto and Varella, 2021) and reported higher level of anxiety during the current COVID-19 pandemic (Semenova et al., 2021).

However, the SARS-CoV-2 virus seems to evade some of the evolved contagion-avoidance adaptations of humans (Ackerman et al., 2020; Seitz et al., 2020; though see Krams et al., 2020, 2021), because infected individuals are contagious before manifesting any symptoms (Tindale et al., 2020), not all infected individuals will manifest any symptoms (i.e., they are asymptomatic) (Cheng et al., 2020; Huff and Singh, 2020), not all symptoms will be present in every infected symptomatic individual, not all symptoms will be easily detectable (e.g., fever, fatigue), the majority of infected symptomatic individuals will develop only a mild version of COVID-19 and will survive, and the incapacitation or death of those few individuals with severe COVID-19 cases will only occur a few weeks after contagion (Grant et al., 2020). All this creates a natural evolutionary mismatch in which the input of a disease necessary to trigger the pathogen avoidance response in others is attenuated or absent (Fraser et al., 2004; Ackerman et al., 2020; Seitz et al., 2020). Further, the hospitalization, cremation, and burials in urban settings do not occur in areas where most people could easily go and verify the numbers of severe cases and deceased first-hand. This adds a layer of human-made mismatch to this domain. Probably, this mostly natural but also human-made evolutionary mismatch of attenuated or absent input within the contagious disease avoidance domain (#1) is responsible for a portion of the current non-compliance with safety measures and transmission-mitigating rules (cf. Fraser et al., 2004; Gandhi et al., 2020). This is further complicated by the tendency in which people affiliate and seek social contact even more when exposed to a threat (Dezecache et al., 2020).

For the majority of individuals who do their utmost to avoid exposure to the virus during the current COVID-19 pandemic (Makhanova and Shepherd, 2020; Nzaji et al., 2020), it has been individually undesirable to stay socially isolated at home (cf. Dezecache et al., 2020; Hwang et al., 2020), advantageous for survival, but at the same time disadvantageous for short-term mate search because it decreased their nocturnal activities (e.g., bars, discos, and nightclubs) (cf. Mannion et al., 2009; Ponzi et al., 2015b; Díaz-Morales et al., 2019). These conflicting domains of adaptive problems comprise an adaptive metaproblem (Al-Shawaf, 2016; Rantala et al., 2019). It is possible that among other factors, such as sex, age, and life history strategy, the level of individual nocturnal tendencies and disease avoidance are moderating factors in determining whether or not an individual trades off mating for survival (or vice versa) during this pandemic (cf. Corpuz et al., 2020; Makhanova and Shepherd, 2020). On the other hand, without conspicuous symptoms and first-hand experiences of COVID-19 outcomes triggering disease avoidance mechanisms (Ackerman et al., 2020; Gandhi et al., 2020; Seitz et al., 2020), most individuals who are less anxious about contagion might simply try to continue their normal lives even in modern pandemic times. For them, there will be no conflicting adaptive problem because the disease-aversion domain would be less or not at all activated.

### Mismatch #2: Artificial Lighting Extending the Evolved Eveningness in Contemporary Life

The evolved propensities of evening-oriented individuals would have long been ancestrally adapted and still currently adaptive under normal nocturnal circumstances (Piffer, 2010; Putilov, 2014). However, since the late industrial age after the human-made diffusion of artificial lighting there has been a further nocturnalization of Western life (Koslofsky, 2011; Vollmer et al., 2012; Rantala et al., 2021). This is further intensified by the screen devices that keep us eagerly focused after sunset and the new wave of blue led lights that strongly suppress the endogenous production of melatonin, the sleep hormone (West et al., 2011; Vollmer et al., 2012; Walker et al., 2020). Thus, it is a case of a human-made, hijacked, input substituted and intensified (because the sunlight that naturally inhibits melatonin is being substituted by artificial light and intensified in the case of blue led lights), individually desirable, and, possibly, fitness-enhancing evolutionary mismatch (#2) (though see Rantala et al., 2021, for the maladaptive outcomes associated with this mismatch).

### Mismatch #3: Virus-Mitigating Social Restrictions Disrupting Evolved Social Life; Evening-Oriented Individuals More Likely to Resist in Favor of Their Ancestral Evolved Tendencies

During the current pandemic, there has been another new human-made environmental change that disrupted normal daily and nocturnal activities: the forced official epidemiologically informed protocol for mitigating viral transmission and for providing public health guidance: beware of the virus and the disease, avoid social gatherings, control your hand movements, maintain improved hygiene, cleanliness, and increase interpersonal space, shelter at home, ventilate indoor settings, avoid multiple intimate contacts with people beyond your close social circle, wear a face mask when outside in order to protect others, and care for the safety of vulnerable ones (cf. Centers for Disease Control and Prevention [CDC], 2020). Although humans have an evolved dispositional tendency to avoid touching or getting in close proximity with sick individuals and to socially distance and attend to hygiene in times of outbreaks (cf. Hewlett and Hewlett, 2007; Ackerman et al., 2018), the specificities of the SARS-CoV-2 virus do not fully trigger psychobehavioral pathogen avoidance mechanisms (the #1 mismatch) (Ackerman et al., 2020; Gandhi et al., 2020; Seitz et al., 2020). Moreover, in ancestral times there were probably no requirements for using face masks, or soap, nor high-concentration alcohol for disinfection during outbreaks, not even the knowledge of why those measures are crucial to mitigate viral transmission and decrease avoidable death rates. Thus, even though there is now almost a year of evolutionarily novel societal protocols used to curb the spread of the SARS-CoV-2 virus, evening-oriented individuals, those who are highly interested in making new social contacts during the night, and those with fast life history strategies may be inclined to continue behaving according to their evolved predispositions (Piffer, 2010; Putilov, 2014; Corpuz et al., 2020; Luoto and Varella, 2021), constituting an evolutionary mismatch (cf. Li et al., 2018; Ackerman et al., 2020). This is the current culturally enacted, human-made, individually undesirable, but fitness-enhancing evolutionary mismatch (#3) that quite possibly made evening-oriented individuals a dangerous liability against epidemiological control. This is because of the previously described mismatches (#1 and #2), and because the core message of the pandemic public health guidance virus-mitigating rules (i.e., focus on survival and refraining from social contact; Centers for Disease Control and Prevention [CDC], 2020; World Health Organization, 2020) clashes with the eveningness profile (cf. Fabbian et al., 2016), which is related to exhibiting paranoia (Sheaves et al., 2016) (paranoia is positively related to conspiracy beliefs, Darwin et al., 2011; Imhoff and Lamberty, 2018), risk-taking (Ponzi et al., 2014), sensation-seeking and impulsivity (Russo et al., 2012; Antúnez et al., 2014), extraversion (Lipnevich et al., 2017), unrestrained hedonism (e.g., Millar et al., 2019), low self-control (Digdon and Howell, 2008), present orientation (e.g., Borisenkov et al., 2019), alcohol, cigarettes, and substance use in public places together with others (Patterson et al., 2016; Millar et al., 2019), multiple short-term mating tendencies (e.g., Díaz-Morales et al., 2019), fast life history (e.g., Marvel-Coen et al., 2018), rule-breaking tendencies (Merikanto et al., 2017), and self-centered remorseless exploitation of others (Jonason et al., 2013), thus trading survival and health-concern for prioritizing sensation-seeking and mating opportunities. During the current pandemic, on average, the evening-oriented profile might put the population at risk by engaging in acts, exhibiting attitudes, and creating contexts that promote and sustain human-to-human contagion.

### Mismatch #4: Pre-pandemic Culturally Imposed Morning-Orientation Leading to Social Jetlag in Evening-Oriented Individuals

**FIGURE 1:**
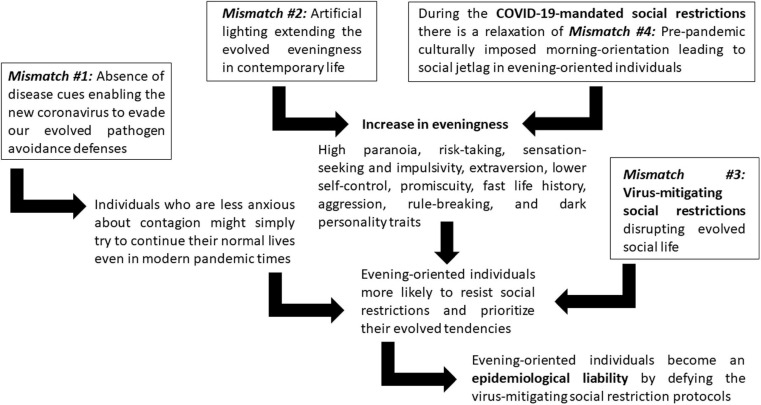
The relationship among the four mismatches and their consequences in relation to the eveningness epidemiological liability hypothesis.

Interestingly, the same virus-mitigating COVID-19-mandated social restrictions might have diminished the human-made culturally induced evolutionary mismatch of having schools, universities, and workplaces biased toward the morning rather than the evening part of the day (#4). This social time pressure mismatch has created a social jetlag in which evening-oriented individuals exhibit massive sleep deficit negatively influencing their bodily and mental health (Korman et al., 2020). However, the COVID-19-mandated social restrictions have relaxed the social time pressure related to social jetlag so individuals can sleep longer and later. A study on 25,000 Argentinians (Leone et al., 2020), another cross-national study on 3,787 participants (Rome et al., 2020), another study on 7,517 participants from 40 countries (Korman et al., 2020), and another cross-national study involving 11 countries on 14,000 participants (Roitblat et al., 2020) showed that COVID-19 related lockdowns and social restrictions have themselves induced significant delays in chronotype. The current weakening of mismatch #4 (morning social pressure) plus the increase of mismatch #2 (artificial lighting) may have converged in increasing evening orientation, which might further complicate the epidemiological liability of mismatch #3 (nocturnal individuals defying the virus-mitigating social restriction protocols). Figure 1 summarizes the connections between the four mismatches and their consequences as the conceptual foundation for the eveningness epidemiological liability hypothesis.

### Four Mismatches Underlie the Eveningness Epidemiological Liability Hypothesis

This sequence of four mismatches culminating in the incongruence between evening-type and compliance with virus-mitigating safety protocols is of crucial relevance for promoting new ways of convincing night-owl individuals to comply with the best practices of pandemic control. The literature on the evolution of disease transmission modes, such as vertical (mother to offspring), or horizontal (sexual, non-sexual/direct contact, airborne, environmental/water/food, fomites/objects, and vector-borne) (Antonovics et al., 2017) should incorporate individual variation in chronotype. Arguably, pre- or asymptomatically contagious infectious diseases (cf. Fraser et al., 2004; Ackerman et al., 2020) which are disseminated by air, direct contact, or sexual activities might create a mismatch in which evening-oriented individuals would be a greater liability against epidemiological control protocols (mismatch #3). If this situation were recurrent enough in the ancestral environment, after higher infections rates and stronger natural selection acting on evening-oriented individuals, we would expect to find in evening-oriented individuals a high evolved resistance against those pathogens using the modes of transmissions that make evening-oriented individuals easier targets for being more promiscuous (e.g., Matchock, 2018; Díaz-Morales et al., 2019) and gregarious (Lipnevich et al., 2017) than morning-oriented individuals, for instance. In contrast, pre- or asymptomatically contagious infectious diseases transmitted by water or mosquitoes’ bites would affect evening-oriented individuals less or equally as morning-oriented individuals. Thus, because not all ancestral disease outbreaks were spread through airborne transmission or through sexual intercourse (e.g., STDs), or had mostly asymptomatic cases, and because there was no precise means or know-how on virus-mitigating measures, evening-oriented individuals were not always a liability against epidemiological control ancestrally. Nevertheless, during the current pandemic, these factors converge leading to the eveningness epidemiological liability hypothesis.

## Eveningness Epidemiological Liability Hypothesis

In fact, a growing body of research has shown that individual traits interfering with social distancing and anti-contagion measures are typical of the nocturnal type. Research on personal factors related to non-compliance with the pandemic safety best practices are still in the initial phases, but it is already possible to see an emerging pattern. The COVID-19 health rule-breakers, i.e., those contributing to non-compliance with the epidemiological safety guidance during the new coronavirus pandemic, tend to be males (Coroiu et al., 2020; Galasso et al., 2020; Nivette et al., 2020; Pollak et al., 2020; Sobol et al., 2020; Tomczyk et al., 2020), exhibit higher proneness to paranoia (Kowalski et al., 2020), and conspiracy beliefs (Biddlestone et al., 2020; Kowalski et al., 2020; Romer and Jamieson, 2020), show higher risk-taking (Miguel et al., 2020; Pollak et al., 2020; Zirenko et al., 2020), tend to not fear the virus, have low perceived risk of COVID-19 (Harper et al., 2020; Pollak et al., 2020), exhibit low self-control (Boylan et al., 2020; Nivette et al., 2020), use more alcohol and drugs (Taylor et al., 2020), tend to be smokers (Pollak et al., 2020), tend to be extraverted (Götz et al., 2020), tend to perceive more mating opportunities (e.g., potential sexual or romantic partners) (Zajenkowski et al., 2020), not have children (Pollak et al., 2020), have faster life history strategies (Corpuz et al., 2020), lower agreeableness (Asselmann et al., 2020; Zajenkowski et al., 2020), lower empathy (Miguel et al., 2020; Zirenko et al., 2020), higher levels of callousness and deceitfulness (Miguel et al., 2020), antisocial potential and moral disengagement from prevention rules (Nivette et al., 2020), higher psychological entitlement (i.e., high expectations for good outcomes, a lack of concern about others, and a distrust of authority figures) (Zitek and Schlund, 2020), and higher Dark Triad personality traits (i.e., Machiavellianism, psychopathy, and narcissism) (Nowak et al., 2020; Zajenkowski et al., 2020; Zirenko et al., 2020).

During the COVID-19 pandemic, non-compliant individuals also tend to be younger (i.e., adolescents and young adults) (Coroiu et al., 2020; Park et al., 2020; Tomczyk et al., 2020). There is ontogenetic variation in human chronotype: evidence indicates a shift toward later chronotype (i.e., higher eveningness) during adolescence, reaching a peak in eveningness at around 19 years, then shifting back toward earlier chronotype (i.e., morningness) thereafter (Randler, 2016; Fischer et al., 2017). Individuals non-adherent to the preventive measures of the pandemic tend to have high levels of ADHD and high psychological distress (Pollak et al., 2020). Evening-oriented individuals are more represented among those with mental disorders (e.g., anxiety, depression, psychosis, and bipolar) and are in general associated with higher psychological distress and symptom severity (Fares et al., 2015; Fabbian et al., 2016; Jones et al., 2016, 2019; Lane et al., 2016; cf. Rantala et al., 2021). Table 1 displays a case-by-case comparison between each facet of the eveningness profile and the non-compliance profile.

**TABLE 1:** Comparison between the eveningness profile and the profile of those who are non-compliant with the virus-mitigating pro-health measures.

Eveningness profile	Non-compliant profile
High in males (Fischer et al., 2017; Piffer, 2010)	Often males (Coroiu et al., 2020; Galasso et al., 2020; Nivette et al., 2020; Pollak et al., 2020; Sobol et al., 2020; Tomczyk et al., 2020)
Peak in eveningness at around 19 years (Randler, 2016; Fischer et al., 2017)	Tend to be adolescents and young adults (Coroiu et al., 2020; Park et al., 2020; Tomczyk et al., 2020)
High symptoms of paranoia, hallucinations (Sheaves et al., 2016)	High proneness to paranoia (Kowalski et al., 2020), and conspiracy beliefs (Biddlestone et al., 2020; Kowalski et al., 2020; Romer and Jamieson, 2020)
High risk-taking (Ponzi et al., 2014), sensation-seeking, and impulsivity (Russo et al., 2012; Antúnez et al., 2014)	High risk-taking (Miguel et al., 2020; Pollak et al., 2020; Zirenko et al., 2020), do not fear the virus, have low perceived risk of COVID-19 (Harper et al., 2020; Pollak et al., 2020)
High extroversion and openness to experience (Lipnevich et al., 2017)	High extroversion (Götz et al., 2020)
Low self-control (Digdon and Howell, 2008; Milfont and Schwarzenthal, 2014), high impulsiveness (Cross, 2010), high present-orientation (Nowack and Van Der Meer, 2013; Stolarski et al., 2013; Borisenkov et al., 2019), high use of alcohol and club/party drugs (Millar et al., 2019), and tend to smoke often (Randler, 2008; Wittmann et al., 2010; Patterson et al., 2016)	Low self-control (Boylan et al., 2020; Nivette et al., 2020), high use of alcohol and drugs (Taylor et al., 2020), tend to be smokers (Pollak et al., 2020)
Fast life history strategy (Ponzi et al., 2015a, b; Marvel-Coen et al., 2018), high promiscuity (Cross, 2010; Piffer, 2010; Ponzi et al., 2015b; Matchock, 2018; Díaz-Morales et al., 2019), have first intercourse at an earlier age (Kasaeian et al., 2019)	Fast life history strategy and promiscuity (Corpuz et al., 2020), high perception of mating opportunities (e.g., potential sexual or romantic partners) (Zajenkowski et al., 2020)
High tendencies toward aggression (Schlarb et al., 2014), rule-breaking behavior (Merikanto et al., 2017), and Dark Triad personality traits, that is, Machiavellianism (e.g., exploitative behaviors), narcissism (e.g., self-orientation), and subclinical psychopathy (e.g., lack of empathy and remorse) (Jonason et al., 2013)	Low agreeableness (Asselmann et al., 2020; Zajenkowski et al., 2020), low empathy (Miguel et al., 2020; Zirenko et al., 2020), high levels of callousness and deceitfulness (Miguel et al., 2020), have antisocial potential and moral disengagement from prevention rules (Nivette et al., 2020), high psychological entitlement (i.e., high expectations for good outcomes, a lack of concern about others, and a distrust of authority figures) (Zitek and Schlund, 2020), high Dark Triad personality traits (i.e., Machiavellianism, psychopathy, and narcissism) (Nowak et al., 2020; Zajenkowski et al., 2020; Zirenko et al., 2020)
High prevalence of mental disorders (e.g., anxiety, depression, psychosis, and bipolar), and high psychological distress and symptom severity (Fares et al., 2015; Fabbian et al., 2016; Jones et al., 2016, 2019; Lane et al., 2016)	High levels of ADHD and high psychological distress (Pollak et al., 2020)

Aligned with the similarities between the evening-oriented profile and the non-complier profile, some of the social contexts of high contagion risk (and situations that have been identified as superspreading events) occur at night. For instance, in Hong Kong the largest cluster of transmission network, the ‘bar and band’ cluster, was traced back to a series of bars totaling 106 infected individuals; for comparison, daytime transmission events, such as a wedding cluster and a temple cluster, had far fewer infections (22 and 19 infected people, respectively: Adam et al., 2020). In Chicago, full-service restaurants had the largest impact on infections, consecutively followed by fitness centers, cafes, and snack bars, hotels and motels, limited-service restaurants, and religious organizations (Chang et al., 2020). In Madison, a cluster with 20 bars had a visitation rate that was positively related to infection rates, and was larger than that of a cluster of 68 restaurants (Harris, 2020). In Vietnam, among other superspreading events, Chau et al. (2020) reported a superspreading event in a bar. In South Korea, among other instances of superspreading situations, Kang et al. (2020) traced back 96 primarily infected individuals from the Seoul nightclubs, and consequently, another 150 secondary and subsequent infections. Among other possibilities, such as private social gatherings or workers living in close quarters, outbreaks in Florida, Texas, California, the Montréal metropolitan area, and in Spain (Catalunya and Aragón) were also linked to the reopening of indoor dining, bars, and nightclubs (Althouse et al., 2020). Although the nocturnal superspreading events are not the only instances of such events, this evidence from across the world strengthens the case for a common nocturnal core of non-compliance during this pandemic.

Moreover, because there is no sunlight and it is colder during night-time, the virus has better survival conditions: it can survive in aerosols more than 2 h at night or indoors, while surviving only 4.8 min at 40°C, 20% humidity, and high intensity sunlight equivalent to noon on a clear summer solstice day at 40°N latitude (Dabisch et al., 2020). This line of evidence connects both previous lines of evidence and strengthens the case for considering the importance of eveningness chronotype as a potential epidemiological liability during the COVID-19 pandemic.

**FIGURE 2:**
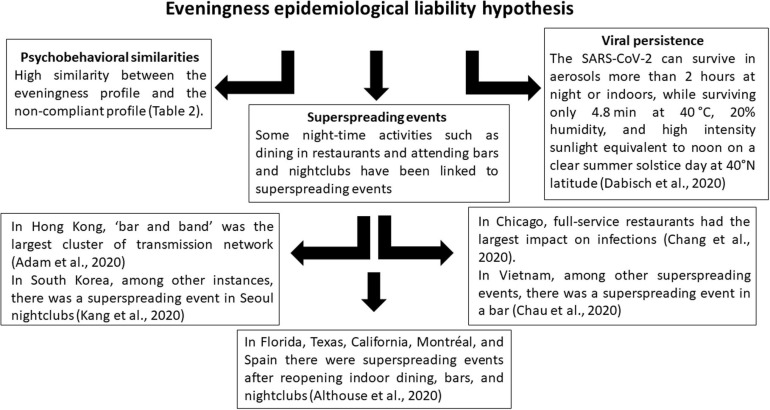
Three sources of available evidence supporting the eveningness epidemiological liability hypothesis.

In sum, the psychobehavioral profiles of those non-compliant with health measures are strikingly similar to those of evening-oriented individuals, with both profiles aligned with fast life history strategies and a neglect of health measures and precautions (Arnot et al., 2020; Corpuz et al., 2020). Some night-time activities such as dining in restaurants and attending bars and nightclubs have been linked to superspreading events; at night there is less health measure enforcement; and at night as well as indoors, the virus has environmental conditions enabling its persistence in aerosol for longer than outdoors during the day. Figure 2 summarizes the three different sources of evidence so far supporting the eveningness epidemiological liability hypothesis. This confluence of factors suggests that epidemiologists, behavioral scientists, psychologists, and policy-makers seeking to minimize the spread of the virus should carefully consider the eveningness chronotype and night-time activities.

## Discussion

The eveningness epidemiological liability hypothesis presented here connects disparate strands of research guided by an evolutionary mismatch approach and life history theory (cf. Ackerman et al., 2020; Corpuz et al., 2020; Seitz et al., 2020), highlighting the possibility that there is a nocturnal psychological core underlying the disposition toward non-compliance to virus-mitigating safety measures during the COVID-19 pandemics. We have presented the evolutionary timeline and context in which, after the habitual use of fire, the consequent expansion of nocturnal activities has amplified some psychological traits in our species, which are related to solving the key adaptive problems occurring nocturnally: its inherent dangerousness, peak tiredness, and easy concealment of identity. Those adaptive challenges have selected for nocturnal individuals to be more paranoid, brave, impulsive, promiscuous, rule-breaking, and gregarious yet antisocial. This fast life-history profile (cf. Marvel-Coen et al., 2018; Arnot et al., 2020), which trades survival and health for prioritizing short-term mating opportunities directly clashes with the core message of the pandemic public health protocols (e.g., Corpuz et al., 2020): focus on survival and refrain from social contact. In the current modern globalized and urbanized context, the increase of use of artificial light after dawn and the relaxation of social time pressure for early activity after pro-health mandatory protocols for social restriction both have contributed to nocturnalization of individuals (West et al., 2011; Vollmer et al., 2012; Korman et al., 2020; Leone et al., 2020; Rome et al., 2020), which may have further complicated the epidemiological liability of evening-oriented individuals as non-compliers. Although cross-culturally population density is not related to COVID-19 infection rates (Krams et al., 2021), the potential anonymity provided by large metropolitan cities that ‘never sleep,’ in addition to the easy individual mobility afforded by such cities when not in an enforced lockdown and/or curfew, might further facilitate the nocturnal transmission which we hypothesize is primarily driven by evening-oriented individuals.

The similarities between the non-complier profile and the evening-oriented individual profile are salient though not complete. For instance, the personality trait openness to experience is related to eveningness (Lipnevich et al., 2017), but it is not related to non-compliance (Asselmann et al., 2020; Zajenkowski et al., 2020), while agreeableness exhibits no relationship with chronotype (Lipnevich et al., 2017), but has appeared as negatively related to non-compliance (Asselmann et al., 2020; Zajenkowski et al., 2020). Future studies focusing on the Big Five personality traits should be able to settle this point.

We have shown that the chronotype is a relevant aspect of (non)compliance in pandemics and that future studies should explicitly focus on it; however, one may counterargue that it is a mere byproduct of sex, age, and life history strategy. Although sex (i.e., males), age (i.e., young individuals), and life history strategy (i.e., fast life history) tend to exhibit a similar profile to the non-complier, we argue that the eveningness chronotype still holds as an important factor. Indeed, the eveningness chronotype is related to male sex, younger age, and fast life history. Further studies should verify which of those variables maintain a unique relationship with non-compliance when the other variables are co-present as predictors. Evening orientation relates to more facets of non-compliance (e.g., includes extraversion), while some aspects of non-compliance (i.e., wearing face mask) do not show sex differences (Howard, 2020) typical of life history. Importantly, the positive relationship between eveningness and rule-breaking tendency holds independently of sex (Merikanto et al., 2017), and some other traits shared with non-compliant individuals are related to chronotype regardless of sex and/or age (e.g., present orientation/instant gratification, Milfont and Schwarzenthal, 2014; mating orientation, Ponzi et al., 2015b; and Dark Triad personality, Jonason et al., 2013). Moreover, some sex differences expected by life history theory (e.g., sex differences in sensation seeking) only appear in evening-oriented individuals (Antúnez et al., 2014). Furthermore, eveningness matches the time period of social contexts in which there are important superspreading events (e.g., dining restaurants, bars, nightclubs), and matches the period of the day in which the virus is more persistent in the environment.

The more the literature on the new coronavirus pandemic develops, the more certainty we can have about this association and its nuances. There are already many preprints out and more research ongoing, which has not yet been peer-reviewed, and hence, not yet included in our literature review. One study reported that compliance with malaria chemoprophylaxis among soldiers during missions in Africa was negatively associated with eveningness chronotype (OR = 0.68) (Resseguier et al., 2010). Another study found that nighttime curfew indeed decreased the acceleration of the new coronavirus propagation, but mostly among the older population and not among the youngest individuals (up to 19 years old), and not as much as the full lockdown (Baunez et al., 2020). Future studies should directly test the eveningness epidemiological liability hypothesis using correlational and mediational approaches and disentangling the effects of eveningness chronotype from those of sex and age, and potentially even those of fast life history strategy. Until then, we argue that the eveningness chronotype should figure alongside the male sex, younger age, and faster life history strategy (Corpuz et al., 2020) as another crucial factor to be studied in the context of non-adherence to pandemic public safety measures.

The eveningness epidemiological liability hypothesis should not lead to the impression that evening-oriented individuals are always a liability during disease outbreaks (i.e., epidemics and pandemics), nor that night-owls have only negative/undesirable psychological traits. The evening-oriented chronotype also exhibits some desirable features such as intelligence (e.g., Stolarski and Jankowski, 2015; Randler, 2017), openness to experience (Randler et al., 2017), and creativity (Giampietro and Cavallera, 2007), and it is only one of many risk-factors for non-compliance. Moreover, a disease with a different mode of transmission might not be related to chronotype at all. Eveningness is therefore not an *a priori* characteristic of pandemic rule-breaking individuals. Furthermore, some evening-oriented individuals may be healthcare workers and thus can better adjust to the night-time work shift, enabling them to keep saving lives while others cannot function properly (e.g., Silva et al., 2017; Hittle and Gillespie, 2018). However, sleep deprivation interferes with immunity and self-regulation (Shields et al., 2017; Rantala et al., 2021), which might increase the risk of contamination with the new coronavirus, increasing shift workers’ vulnerability especially in the healthcare frontline tackling the pandemic (Silva et al., 2020). Thus, not all evening-oriented individuals are a liability or non-compliant with the pandemic safety measures.

### The Bigger Picture in COVID-19 Non-Compliance

Far from being the definitive all-encompassing explanation, the eveningness epidemiological liability hypothesis, which focuses more on individual dispositional traits, is only a part of the bigger picture. That is because there are multiple factors in different domains and levels influencing (non)compliance with the health guidelines during the COVID-19 pandemic. Political orientation, such as authoritarianism, is related to low frequency of face mask wearing in public (Prichard and Christman, 2020). Further, situational and contextual factors also play a role. Situational predictions of non-compliance are: unemployment status, being the woman head of a family, and having less information and low-quality knowledge about COVID-19 (Nzaji et al., 2020). Low exposure to the instructions is also related to non-adherence to safety measures (Pollak et al., 2020), as it is for media diets (Pedersen and Favero, 2020). Some research has even demonstrated that situational factors explained more variance in compliance than dispositional personality traits (Zajenkowski et al., 2020). Most of the abovementioned situational factors—that is, knowledge about the disease and the quarantine, social norms, perceived benefits of quarantine and risks of the disease, lack of supplies, and unemployment—are associated with (non)adherence to quarantine during infectious disease outbreaks (Webster et al., 2020). Moreover, societal norms (loose vs. tight) may also contribute to the varying reactions of world nations in limiting COVID-19 cases and deaths (Seitz et al., 2020; Gelfand et al., 2021). Hence, males, young individuals, those pursuing a fast life history strategy, and evening-oriented individuals are not the only non-compliant type(s); because of situational reasons, many more individuals also end up defying the contagion-mitigating measures. This polymorphic nature of non-compliance suggests public policies should have many profile targets, since “one size fits all” policies are unlikely to be as effective (cf. Arnot et al., 2020). Future studies should always access and control for both dispositional/internal traits (e.g., personality and life history) and situational/external factors (e.g., employment status, marital/relationship status) in order to establish a more comprehensive picture of the phenomenon of non-compliance with virus-mitigating measures (cf. Zajenkowski et al., 2020).

The message is similar regarding superspreading events. The superspreading events emphasized here were all characterized by the “opportunistic” type, in which individuals temporarily cluster in crowded places and sing or speak loudly (Althouse et al., 2020). However, there are other types of superspreading events: the “biological,” in which some individuals have an unusually high viral load; the “behavioral/social,” in which some individuals have an unusually high number of daily social contacts; and the “high-risk facilities and places,” in which individuals are repeatedly exposed to higher potential infection rates such as meat-packing plants, workers’ dormitories, prisons, long-term care facilities, or healthcare settings (Althouse et al., 2020). Future studies should sample widely from all those types of events. However, one of the main differences between the opportunistic and the other types is that the former is normally an individual choice done during leisure time, while the others are somewhat more difficult to avoid without imposing extensive societal lockdowns. Therefore, places and contexts in which “opportunistic” superspreading events have occurred are in theory a good target for public communication convincing individuals to avoid those same places during pandemics for safety reasons, or public policy interventions restricting such events altogether.

Further, the methods to quantify non-adherence to public health measures vary across studies and are not yet standardized and validated measures. In the same way that laboratories are rushing to get viable vaccines, other researchers are rushing to create new methods to define and address non-compliance with pandemic health measures. Some studies focus on intentions to follow public health guidelines while others focus on actual or past behavior. Some studies use questionnaires, some use direct observation about actual behavior in public settings, others use GPS data from public cellphones to track individual movements. Some studies focus only on one type of public health safety measure, such as shelter in place, or mask-wearing, while others access a variety of measures from hand sanitizing to avoiding crowded places. The way to define who is non-compliant also varies across studies: some use a histogram-informed cut in the scale used, while others rely mostly on whether people have left home in the last 24 h. This is an expected problem given how recent the pandemic and, consequently, all this related research is. As the second and subsequent waves of the COVID-19 pandemic unfolds (Krams et al., 2021), future studies should invest into unifying and validating measures of non-adherence to pro-health measures in order to promote more comparability among different studies in different places of the world, and to increase certainty about the actual scientific value of each quantitative method employed to study non-compliance with safety measures. A mixed or multi-method approach is also desirable because different methods complement each other in their strengths. Further, a globally distributed collaborative network of laboratories could be decisive to improve the representativeness and the power of studies on pandemic (non)compliance (cf. Moshontz et al., 2018).

### Future Research

Nevertheless, we argue that the available evidence stemming from different sources and places around the world is convergent and strong enough to indicate a distinctive pattern: that evening-oriented individuals are probably making a significant contribution to the continuous spreading of the new coronavirus, extending and worsening this pandemic and public health crisis.

Further studies in different populations are needed to establish the veracity of this hypothesis, the size of the effect, and the extent of its validity across different nations and contexts. One possible way of testing the eveningness epidemiological liability hypothesis is checking whether the eveningness chronotype indeed positively correlates with non-compliance with the virus-mitigating health protocols of the current pandemics (e.g., reduced use of shelter in place, no face mask wearing in public, low avoidance of indoor crowded places, etc.), even controlling for sex, age, life history strategy, political orientation, and possibility of home office. In a path analysis design, researchers could test whether any of the following traits, such as paranoia, risk-taking, sensation-seeking and impulsivity, extraversion, lower self-control, promiscuity, aggression, rule-breaking, and dark personality traits act as moderating variables between chronotype and non-compliance. We would predict these traits to be positively correlated with non-compliance. Another possibility is to investigate whether evening-oriented individuals have been infected more frequently by the new coronavirus (i.e., SARS-CoV-2) than morning-oriented individuals. Studies that access cell phone GPS big data to infer individual mobility can analyze whether places with more nocturnal activities have higher infection rates. Epidemiological studies could compare locations before lockdown with and without night-time curfew to analyse how much of the viral transmission is decreased by impeding nocturnal activities.

### Implications for Public Policy

Until then, on the safe side, it is appropriate after careful consideration (IJzerman et al., 2020) to promote security measures that take the crucial sequence of evolutionary mismatches and adaptive metaproblem into account and focus on how to avoid nocturnal individuals from becoming superspreaders while offering secure alternatives for their nocturnal activities. These can include online substitutive activities (e.g., live streams instead of music concerts, social media/video calls instead of in-person socialization, Netflix/movie streaming platforms instead of cinema, online dating instead of flirting in bars, virtual sexual activities instead of in-person intercourse, etc.). Virtual reality technology, which has significant applications in times of COVID-19 pandemic (Singh et al., 2020), also should be used to offer secure social alternatives to evening-oriented individuals.

Our first suggestion is to take chronotype into consideration alongside sex and age as important factors influencing the effectiveness of public communication about pro-health measures. Tailoring the messages to appeal to this psychobehavioral profile could be a very effective strategy; however, caution needs to be taken when applying behavioral research to public policy (IJzerman et al., 2020). In Japan, Nakayachi et al. (2020) discovered that people conformed to societal norms in wearing masks, i.e., the more they heard that most people were using masks, the more willing they became to use them. For instance, one option would be to use this strategy geared toward night-owls, males, and youngsters. Other social psychological and behavioral strategies and techniques (cf. Kantor, 2020; Smith and Gibson, 2020; Van Bavel et al., 2020) might be directed toward predominant evening-types, males, and youngsters in order to minimize viral transmission.

The second suggestion arising from this review is to limit access to those places where evening-oriented individuals normally go during night-time (e.g., bars, nightclubs). Haug et al. (2020) ranked the effectiveness of government non-pharmaceutical interventions worldwide to curb the COVID-19 pandemic: the most effective measures comprised closing and restricting most places where individuals tend to gather in smaller or larger numbers for extended periods of time, such as schools, businesses, and bars. Independently, Brauner et al. (2020) did a similar worldwide analysis and concluded that closing some businesses such as restaurants, bars, nightclubs, cinemas, and gyms had a moderate-to-small effect in reducing COVID-19 transmission, indicating a promising policy option together with limiting gatherings to 10 people or less, which exhibited a large effect in reducing COVID-19 transmission. In France, nighttime curfew indeed decreased the acceleration of SARS-CoV-2 transmission, but the subsequent lockdown was more effective (Baunez et al., 2020). Hence, it seems that a night-time shutdown would indeed inhibit COVID-19 transmission. However, prohibition in some places might also generate clandestine bars and gathering places. Therefore, depending on a low and favorable local transmission rate, some alternative options could remain open, primarily in open air, with good ventilation, in ample spaces, with lower numbers of individuals, all wearing masks. On the other hand, on places where the local transmission rate is rampant due to the new variants of SARS-CoV-2, nighttime curfew is not enough, only a full lockdown, case tracing, and massive vaccination are likely effective.

A consensus reached by a group of experts from the Spanish Association of Sexuality and Mental Health strongly recommended not initiating sexual activity with a sporadic partner during pandemics for safety reasons (Cabello et al., 2020), a recommendation endorsed by Ibarra et al. (2020). Indeed, there has been a decrease in sexual partners, sexual frequency, and sexual risk-taking during the COVID-19 pandemic (Li et al., 2020). Thus, the higher tendency toward casual sex that evening-oriented individuals have can be partially met with virtual sexual activities such as telephone or online sex. This is what the International Society for the Study of Women’s Sexual Health (International Society for the Study of Women’s Sexual Health [ISSWSH], 2020) recommended since May 2020: the new safe sex is ‘e-sex.’ Dating apps still allow people to search for new possible partners and flirt safely online. Even those individuals with steady partners who are under quarantine after testing positive for COVID-19, those with some clinical symptoms, those who are pregnant, and those health professionals who are in contact with COVID-19 patients are recommended to abstain from coital/oral/anal sex, substituting it with masturbatory or virtual sexual activity to provide maximum protection against SARS-CoV-2 contagion (Cabello et al., 2020). The dangers inherent in casual sex can create a delicate situation since the life satisfaction of singles has decreased most during COVID-19 lockdowns (Hamermesh, 2020).

Interestingly, there is a growing related literature pointing out that the circadian rhythms which influences gene expression and many cellular and physiological parameters may influence individual susceptibility and resilience to viral infections which interacts with those circadian internal changes (Diallo et al., 2020; Sengupta et al., 2021). Antiviral therapies in COVID-19 patients are more efficient when provided in the morning as opposed to in the evening (De Giorgi et al., 2020), which is presumably independent from individuals chronotype. This circadian rhythm literature, when connected to the findings on the relaxation of social jetlag during the pandemic (e.g., Korman et al., 2020), should be integrated with the non-compliance literature according to the hypothesis presented here for us to be able to have a clear, broad, and deeper understanding of the many ways in which circadian rhythms/chronotype can influence and be influenced by the dynamics of the current COVID-19 pandemic. Chronotype and circadian rhythms are still missing in the discussion about the psychology of pandemics (cf. Taylor, 2019; Ackerman et al., 2020).

## Conclusion

We have shown a sequence of evolutionary mismatches (Figure 1) and an adaptive metaproblem between the context of the evolved psychological profile for nocturnal activities and the compliance with modern pandemic restriction protocols in the context of an infectious respiratory disease outbreak. The main hypothesis put forth in this article is that evolved propensities for nocturnal activities constitute a liability against proper epidemiological control during the current pandemic. Although still lacking key pieces of evidence and being only a part of the bigger picture of non-compliance (cf. Zajenkowski et al., 2020), this hypothesis and its subsequent empirical testing/falsification constitute an important step toward improving public health communication and effectively targeting campaigns to potential superspreaders.

This *eveningness epidemiological liability hypothesis* is substantiated by connecting three main lines of empirical evidence. First, the SARS-CoV-2 is able to persist in aerosols much longer during the night and indoors than during the day outdoors. This sets the stage for the possibility of relatively higher viral transmission and individual contagion during the night. Second, nocturnal activities such as restaurant dining, bars, and nightclubs were identified as contexts of high contamination risk, even originating some superspreading events across-countries (Figure 2). This confirms and specifies which night-time places and activities individuals are willingly seeking during their leisure time (i.e., those enabling short-term mating) when they are at an elevated risk of being contaminated or transmitting the new coronavirus. The third line of evidence concerns the psychobehavioral profile comparison between evening-oriented individuals and those non-compliant with public health guidelines (Table 1). Importantly, evening-oriented individuals tend to be rule-breakers (Merikanto et al., 2017), which precisely meets the definition of non-compliance with the pandemic safety rules. Both tend to be more frequently males, on average younger, more paranoid, remorseless/antisocial, impulsive, higher in risk-taking, smoking, alcohol use, drug use, extraversion, and short-term mating. These factors are aligned with the fast life-history profile that sexual selection would favor in a nocturnal context of short-term mate search and risky sexual behaviors, but which could maladaptively backfire in a pandemic context of an airborne virus (e.g., Ponzi et al., 2015b; Arnot et al., 2020; Corpuz et al., 2020).

All those same psychobehavioral tendencies amplified in the nocturnal niche throughout human evolution arising via the longstanding habitual use of fire might unfortunately contribute to setting the pandemics ‘on fire’ via increased transmission rates. This is still a hypothesis that requires a systematic empirical test and should not be used as an excuse to persecute evening-oriented individuals nor to justify enforcing nighttime curfew when the situation requires a full lockdown.

We have developed the eveningness epidemiological liability hypothesis with convergent and empirical evidence (e.g., observational, survey, and cross-cultural studies as well as meta-analyses) stemming from a variety of fields, and based it on a plausible and circumstantiated evolutionary analysis that includes consideration of phylogeny, adaptive challenges, evolutionary mismatches, and adaptive metaproblems.

Without the theoretical foundation presented here, any possible study showing a positive correlation between eveningness and non-compliance would come across as just another correlate of non-compliance among many, and not as a possible core feature of the phenomenon. The evolutionary mismatch hypothesis of eveningness epidemiological liability connects disparate strands of evidence, organizes some of the profusion of recent findings, whilst also helping to guide and focus public health preventive measures. We have presented a broad literature review and a new evolutionarily oriented hypothesis, discussed it against possible alternatives (such as sex, age, and life history) at such length that would not be possible in an empirical research article.

We hope this article can motivate other researchers to improve upon and test this framework comprising four mismatches, an adaptive metaproblem, and the eveningness epidemiological liability hypothesis, serving as a heuristic theoretical and hypothesis-generating review for future confirmatory research. As such, we have made it possible to skip the process of conducting exploratory empirical analyses on the topic and guided researchers straight into doing confirmatory analyses on the framework of this article, which in times of pandemics is desirable given the urgency and severity of the global epidemiological, societal, and economic crises.

## Data Availability Statement

This is a purely theoretical contribution, thus there is no data for the authors to make available. The original contribution presented in the study is already included in the article, further inquiries can be directed to the corresponding author.

## Author Contributions

MV conceived and searched the literature and organized and drafted the manuscript. SL, RS, and JV made substantial contributions and reviewed the manuscript critically for intellectual content. MV, SL, RS, and JV revised and approved the final manuscript for submission. All the authors contributed to the article and approved the submitted version.

## Conflict of Interest

The authors declare that the research was conducted in the absence of any commercial or financial relationships that could be construed as a potential conflict of interest.
